# HLA-G: A New Immune Checkpoint in Cancer?

**DOI:** 10.3390/ijms21124528

**Published:** 2020-06-25

**Authors:** Daniëlle Krijgsman, Jessica Roelands, Wouter Hendrickx, Davide Bedognetti, Peter J. K. Kuppen

**Affiliations:** 1 Department of Surgery, Leiden University Medical Center, P.O. Box 9600, 2300 RC Leiden, The Netherlands; d.krijgsman@lumc.nl (D.K.); jroelands@sidra.org (J.R.); 2 Cancer Research Department, Research Branch, Sidra Medicine, Doha P.O. Box 26999, Qatar; whendrickx@sidra.org (W.H.); dbedognetti@sidra.org (D.B.)

**Keywords:** HLA-G, immunotherapy, immune checkpoint, cancer

## Abstract

Human leukocyte antigen G (HLA-G), known as a central protein in providing immune tolerance to the fetus in pregnant women, is also studied for a possible role in tumor development. Many studies have claimed HLA-G as a new immune checkpoint in cancer. Therefore, HLA-G and its receptors might be targets for immune checkpoint blockade in cancer immunotherapy. In order to substantiate that HLA-G is indeed an immune checkpoint in cancer, two important questions need to be answered: (1) To what extent is HLA-G expressed in the tumor by cancer cells? and (2) What is the function of HLA-G in cancer immune evasion? In this review, we discuss these questions. We agree that HLA-G is a potentially new immune checkpoint in cancer, but additional evidence is required to show the extent of intra-tumor and inter-tumor expression. These studies should focus on tumor expression patterns of the seven different HLA-G isoforms and of the receptors for HLA-G. Furthermore, specific roles for the different HLA-G isoforms should be established.

## 1. Introduction

Human leukocyte antigen G (HLA-G) is known as a central protein in providing immune tolerance to the fetus in pregnant women [1,2]. Because of its immune-inhibiting function, it is also studied for its role in tumor development, where it may function as an immune checkpoint. Several immune checkpoints have been identified, which, among others, include programmed cell death protein 1 (PD-1) and cytotoxic T-lymphocyte-associated protein 4 (CTLA-4), of which their ligands (programmed death ligand-1 and -2 (PD-L1/PD-L2) and B7, respectively) can be expressed by tumor cells to escape recognition by the immune system [3]. Blocking the interaction between the molecules involved in immune checkpoint signaling using monoclonal antibodies has led to remarkable therapeutic success in cancer [4]. Many studies have claimed HLA-G as a new immune checkpoint in cancer [5]. Therefore, HLA-G and its receptors might be targets for immune checkpoint blockade in cancer immunotherapy. In order to substantiate that HLA-G is indeed an immune checkpoint in cancer, two important questions need to be answered: (1) To what extent is HLA-G expressed in the tumor by cancer cells? and (2) What is the function of HLA-G in cancer immune evasion? Here, we discuss evidence for possible answers to these two questions. Finally, we propose future directions for research on the role of HLA-G in cancer.

## 2. HLA-G

**Figure 1:**
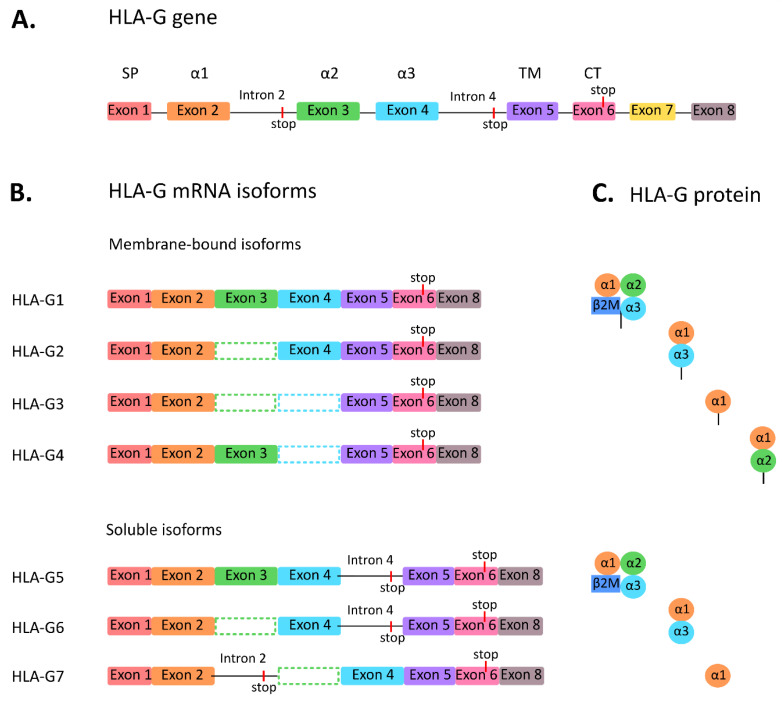
Human leukocyte antigen G (HLA-G) isoforms. (**A**) Overview of the HLA-G gene, (**B**) the seven mRNA splice variants of the HLA-G gene and (**C**) its resulting protein structures. Abbreviations: cytoplasmic tail (CT), signal protein (SP), transmembrane (TM).

The HLA-G gene is located on chromosome 6 at region 6p21.3, within the class I gene cluster of the major histocompatibility complex (MHC). As a result of alternative RNA splicing, seven isoforms can be formed, comprising four membrane-bound isoforms (HLA-G1, G2, G3, and G4), and three secreted soluble isoforms (HLA-G5, G6, and G7) [6,7,8], see Figure 1. Most studies focus on the full-length molecule (HLA-G1) and its soluble counterpart (HLA-G5). These isoforms are identical, except HLA-G5 is missing the transmembrane part. Both are associated with β2-microglobulin (β2M), like the other HLA class I molecules. The other HLA-G isoforms are smaller since they lack globular domains and do not bind β2M. The RNA splice variants of HLA-G have been studied thoroughly, as shown by the many reports in the literature [9,10,11]. However, the question remains whether all splice variants translate into protein. The crystal structures of HLA-G1 and HLA-G2 have been described [12,13], but, to our knowledge, the existence of other HLA-G isoforms has not been confirmed using crystallography. Figure 1 shows the proposed protein composition of HLA-G isoforms based on the presence of specific exons in their mRNA structure.

## 3. Receptors of HLA-G

HLA-G mediates its function by binding to receptors on immune cells. The known receptors are leukocyte Ig-like receptor subfamily B member 1 (LILRB1) and member 2 (LILRB2), also known as ILT2 and ILT4, and the killer immunoglobulin-like receptor 2DL4 (KIR2DL4). The ILT2 receptor recognizes the α3 domain of HLA-G in association with β2M, thereby recognizing the HLA-G1 and HLA-G5 isoforms [12,14]. ILT2 is expressed by all monocytes and B cells, but also by subsets of natural killer (NK) cells, T cells, dendritic cells and myeloid-derived suppressive cells (MDSCs) [15]. Additionally, the ILT4 receptor also recognizes the α3 domain of HLA-G but without its association with β2M [12,14]. ILT4 therefore presumably recognizes the HLA-G2 and HLA-G6 isoforms, but also β2M-free HLA-G1 and HLA-G5, and is mainly expressed by monocytes, neutrophils, dendritic cells and MDSCs [16,17,18]. ILT/HLA-G interaction has been associated with the suppression of immune cells, including the impairment of proliferation, differentiation, cytokine secretion, cytotoxicity, and chemotaxis [19,20,21]. Furthermore, KIR2DL4 recognizes the α1 domain of HLA-G and therefore, presumably, all HLA-G isoforms [22]. KIR2DL4 is expressed by NK cells and a subset of T cells [23,24], and has been shown to induce the inhibition of immune functions of these cells [25,26] as well as activation [27] upon binding with HLA-G. For instance, a study by Rajagopalan et al. reported that soluble HLA-G induced a proinflammatory response via KIR2DL4 in resting NK cells [27]. The heterogeneous expression patterns of the different isoforms of HLA-G [10,28] and their receptors [29,30,31] suggest a fine-tuned network which is closely involved in the regulation of immune interactions. Unfortunately, little is known about the signaling pathways regulating this network. As discussed above, HLA-G isoforms interact with different receptors that are present on distinct immune cell subsets. Therefore, it is likely that HLA-G isoforms might have different functions, including in cancer.

## 4. Expression of HLA-G in Cancer

**Figure 2:**
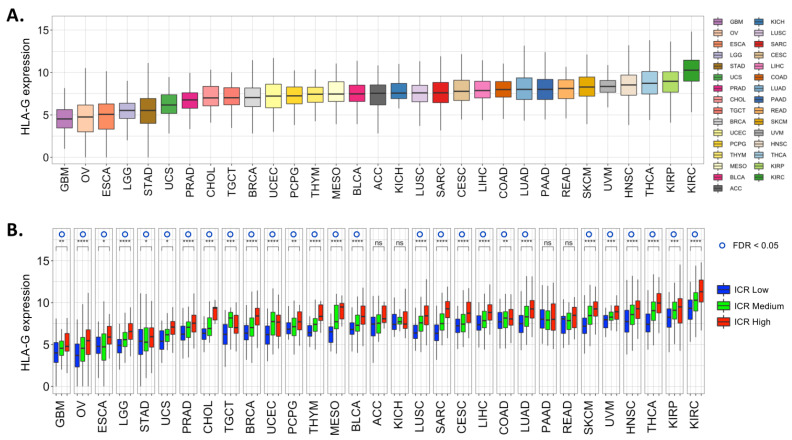
HLA-G mRNA expression in The Cancer Genome Atlas (TCGA) cohort. (**A**) Boxplot of log-transformed pan-cancer normalized gene expression values of *HLA-G* across 31 solid cancer types from The Cancer Genome Atlas [49]. Cancer types are ordered by mean expression of *HLA-G* per cancer type. (**B**) Log-transformed pan-cancer normalized gene expression per Immunologic Constant of Rejection (ICR) cluster for each cancer type. Unpaired *t*-test between *HLA-G* expression in ICR High versus ICR Low: * *p* < 0.05, ** *p* < 0.01, *** *p* < 0.001, **** *p* < 0.0001, and ns: not significant. False Discovery Rate (FDR) < 0.05 by Benjamini-Hochberg method is annotated by blue circles. Immunologic Constant of Rejection (ICR). Glioblastoma multiforme (GBM). Ovarian serous cystadenocarcinoma (OV). Esophageal carcinoma (ESCA). Brain Lower Grade Glioma (LGG). Stomach adenocarcinoma (STAD). Uterine Carcinosarcoma (UCS). Prostate adenocarcinoma (PRAD). Cholangiocarcinoma (CHOL). Testicular Germ Cell Tumors (TGCT). Breast invasive carcinoma (BRCA). Uterine Corpus Endometrial Carcinoma (UCEC). Pheochromocytoma and Paraganglioma (PCPG). Thymoma (THYM). Mesothelioma (MESO). Bladder Urothelial Carcinoma (BLCA). Adrenocortical carcinoma (ACC). Kidney Chromophobe (KICH). Lung squamous cell carcinoma (LUSC). Sarcoma (SARC). Cervical squamous cell carcinoma and endocervical adenocarcinoma (CESC). Liver hepatocellular carcinoma (LIHC). Colon adenocarcinoma (COAD). Lung adenocarcinoma (LUAD). Pancreatic adenocarcinoma (PAAD). Rectum adenocarcinoma (READ). Skin Cutaneous Melanoma (SKCM). Uveal Melanoma (UVM). Head and neck squamous cell carcinoma (HNSC). Thyroid carcinoma (THCA). Kidney renal papillary cell carcinoma (KIRP). Kidney renal clear cell carcinoma (KIRC).

Over the years, many studies have reported on the expression of HLA-G in many different types of cancers [32,33,34,35], usually via evaluation of immunohistochemical staining (IHS) of tumor tissues using HLA-G-recognizing antibodies. These studies showed that many tumors express HLA-G, including, among others, colorectal cancer (CRC), breast cancer, and lung cancer [32,33,34,35]. Although IHS is a widely accepted technique, the detection of HLA-G with IHS is controversial, with expression levels reported in >70% of the evaluated tumors in some studies [36,37], whereas others reported HLA-G expression in only 20% of the evaluated tumors of similar types [33]. These results illustrate that IHS results regarding HLA-G expression should be interpreted with great caution. A part of the discrepancies can be explained by the use of different antibodies that each recognize different epitopes of HLA-G, which may lead to the detection of different combinations of isoforms, and therefore distinct staining patterns. The most commonly used HLA-G-recognizing antibodies are summarized in Table 1 (adapted from [11]). Furthermore, cross-reaction has been reportedly widely used for commercially available HLA-G antibodies such as 4H84 and MEM-G/1 with other HLA class I molecules and with as yet unidentified proteins [38,39,40]. This might result in the overestimation of HLA-G expression in tumors that highly express proteins that are also recognized by HLA-G-recognizing antibodies due to cross-reaction, thereby further contributing to discrepancies between studies. In addition, different cut-off levels are used among studies to define positivity for HLA-G expression, which may also explain the differences between studies. It has been shown that HLA-G expression could be detected by Western blot in IHS-confirmed HLA-G^+^ tumors, like lung tumors [29], but not in all IHS-confirmed HLA-G^+^ primary hepatocellular carcinomas [41] or CRC tumors [40]. Therefore, we concluded that claiming that HLA-G plays a role in intra-tumoral immune modulation seems premature, as the question remains as to what extent HLA-G is really expressed in tumors. This emphasizes that results claiming that tumors express HLA-G should be interpreted with caution. In order to obtain reliable data on HLA-G expression using IHS in future studies, it is of high importance that reliable antibodies are used. This substantiates the need for new HLA-G-recognizing antibodies that do not cross-react with other proteins. Until that is the case, it is essential to use different techniques (for instance, western blotting) in addition to IHS in order to validate HLA-G expression in cancer. Another indication of HLA-G expression, independent of antibodies, could be the level of mRNA expression in tumors. Therefore, we analyzed the HLA-G transcript abundance, reflecting the gene expression of all HLA-G isoforms in aggregate, by mRNA sequencing in primary tumor tissues from The Cancer Genome Atlas (TCGA), a publicly available database, in a pan-cancer normalized expression matrix [42]. The results are shown in Figure 2A and reveal that the HLA-G gene is indeed expressed among different tumor types. Furthermore, RT-qPCR has been used to study HLA-G isoform mRNA expression. For instance, Sarmah et al. reported on HLA-G isoform frequency in 35 head and neck squamous cell carcinoma patients [28]. We did a similar study in CRC and detected mRNA of HLA-G3 in different samples [9]. In addition, we showed that it is probably not DNA methylation of the promoter region of HLA-G that controls mRNA expression [9]. We conclude from these studies and data that it is quite likely that several tumors express HLA-G mRNA. The next question is whether HLA-G mRNA in tumor cells translates to protein. We [9] and others [43] have reported discrepancies between HLA-G mRNA and protein expression in cancer. Recent studies suggested that HLA-G is heavily post-transcriptionally regulated [44,45], in which miRNA may play an important role [46,47]. For instance, a study on renal cell carcinoma showed strong post-transcriptional gene regulation of HLA-G by miRNA-152, miRNA-148A, miRNA-148B, and miRNA-133A [48]. Interestingly, the stable overexpression of miRNA-148A and miRNA-133A in target cells caused the downregulation of HLA-G protein expression, thereby enhancing the NK cell-mediated killing of these cells in vitro. In summary, despite highly variable results, the available data suggest that HLA-G can be expressed de novo by human malignant cells, possibly due to deregulated posttranscriptional mechanisms.

## 5. Function of HLA-G in Cancer

To explore in which context the HLA-G gene is expressed across different cancers with respect to their tumor immune phenotype, we compared HLA-G mRNA expression between “hot” or T-helper 1 type (Th1)-inflamed tumors versus “cold” or immune-silent tumors. Genes that are typically associated with immune-mediated tissue rejection include Th1-related and interferon-stimulated genes, CXCR3/CCR5 chemokine-receptor signaling, cytotoxic effector molecules, and counter-activated immune regulatory genes [49,50,51,52]. An evaluation of the expression of 20 genes that reflect these immunological processes, referred to as the Immunologic Constant of Rejection (ICR), can segregate tumors across ICR Low or “cold”, ICR Medium, and ICR High or “hot” tumors, as described in detail elsewhere [42]. A comparison of HLA-G gene expression between ICR High and ICR Low groups identified the significantly increased expression of HLA-G in the ICR High tumors in most cancer types (unpaired *t*-test, *p* < 0.05; Figure 2B). Previously, a similar observation was made across different independent cohorts of breast cancer patients: HLA-G gene expression was consistently upregulated in ICR High or Th1-inflamed breast tumors [53]. The positive association between the expression of pro-inflammatory transcripts and HLA-G could indicate that HLA-G is a counter-regulatory mechanism that follows the intra-tumoral infiltration of activated lymphocytes. In fact, the expression of “classic” immune checkpoints like programmed death ligand-1 (PD-L1), programmed cell death protein 1 (PD1), cytotoxic T-lymphocyte-associated protein 4 (CTLA-4), and indoleamine 2,3-dioxygenase dioxygenase 1 (IDO1), also has a high correlation with pro-inflammatory transcripts [54,55]. These data indicate that HLA-G may be upregulated in tumors to suppress the tumor-induced immune response. HLA-G may therefore function as an immune checkpoint in cancer, like it does in pregnancy, i.e., to protect against an immune response. This may result in the outgrowth of tumor cells that do express HLA-G.

Several in vitro studies showed that HLA-G-negative leukemia, glioma, ovarian carcinoma, and hepatocellular carcinoma cell lines are more efficiently killed by NK cells compared to their HLA-G transfected counterparts [41,56,57,58]. Interestingly, blocking with HLA-G antibodies restored the NK-mediated lysis of the targeted cancer cell lines, suggesting a role for HLA-G in tumor immune evasion that can be modulated. Additionally, the in vivo role of HLA-G as a tumor immune escape mechanism has been demonstrated in mouse models [20,59,60]. It is important to consider that a murine counterpart of HLA-G does not exist and, therefore, it is debatable whether results from such murine models would translate to the human physiology. In humans, the frequency of HLA-G expression has been widely studied using IHS, and analyzed in relation with clinical outcome. Most studies showed that HLA-G expression in cancer is associated with a poor clinical outcome in patients [32,33,34,35], suggesting that HLA-G plays a role in immune evasion and disease progression. Furthermore, studies reported an association between HLA-G expression and clinical parameters associated with progressive disease in lung cancer, including lymph node invasion, higher disease stage and poor differentiation [29] and a correlation with increased Tumor-Node-Metastasis (TNM) stage in CRC [31]. Based on these results, many studies claimed that HLA-G is an immune checkpoint in cancer (reviewed in [5]). However, there are also studies that suggest the opposite, i.e., that HLA-G expression is associated with a better clinical outcome in patients with ovarian carcinoma and rectal cancer [61,62]. Figure 2 shows increased HLA-G mRNA expression in ICR High tumors in ovarian carcinoma, but not in rectal cancer. Importantly, these data only provide information on a genomic level, but not on a protein level. Furthermore, the high expression of HLA-G in ICR High tumors does not necessarily reflect the role of HLA-G in the clinical outcome of these patients. Importantly, HLA-G may have different roles in anti-tumor immune responses due to the fact that HLA-G isoforms can bind to different receptors that are present on distinct immune cell subsets. To complicate this matter even more, HLA-G^+^ tumor cells have been reported to secrete HLA-G^+^ exosomes, which have been associated with immunosuppression and disease progression in different cancer types (reviewed in [63]). Furthermore, HLA-G^+^ cells have been reported to transfer their HLA-G expression to other cells, including NK and T cells, in a process called “trogocytosis” [64,65,66]. In myeloma patients, HLA-G transfer from tumor cells to T cells via trogocytosis was associated with a poor clinical outcome [65]. It is possible that the functions of HLA-G^+^ exosomes and trogocytosis in cancer are dependent on which isoforms are involved and the cells HLA-G is transferred to. The effect of HLA-G tumor expression on clinical outcome might therefore be highly dependent on (1) the expression pattern of HLA-G isoforms in the tumor (including release of exosomes), and (2) the immune cell composition of the tumor. An alternative explanation is also possible. Perhaps the fact that HLA-G is expressed in tumors may reflect a high level of epigenetic deregulation and/or mutational burden in cancer, correlating with aggressive cancer types in which the immune response is present and activated but not able to combat tumor growth. In other tumors, like the aforementioned ovarian cancer and rectal cancer, where HLA-G is associated with a better clinical outcome, it may also reflect genome integrity rather than a functional relationship with an antitumor response. Therefore, in our opinion, it is premature to state that HLA-G is an immune checkpoint based on the survival data of cancer patients only, since the immune system is highly complex. A study by Rouas-Freiss et al. reported a high intratumor heterogeneity in the renal cell cancer of HLA-G/ILT2/ILT4 and PD-1/PD-L1 expression, showing the complexity of tumor–immune interactions [30]. Up till now, there are, to our knowledge, no studies available that further report on the interaction between tumor-expressed HLA-G and its receptors ILT2, ILT4, and KIR2DL4 on immune cells and resulting effects. Investigation of these interactions, for instance with single cell technology, will be necessary to further explore the role of HLA-G in tumor–immune interactions. Additionally, HLA-G is often studied as if it is one molecule. However, as mentioned above, it is known that at least seven HLA-G isoforms are present, which may all have different functions. There are indications that soluble HLA-G is also expressed and released by cancer cells and, as a consequence, is detectable in the circulation of cancer patients [67,68]. Methods should be further developed to determine the expression of the different isoforms in tumors and to establish their function. For instance, new antibodies need to be developed that recognize distinct HLA-G isoforms in IHS without cross-reacting with other proteins. We feel these are important challenges for the near future.

## 6. Conclusions

HLA-G is a potentially new immune checkpoint in cancer, but additional evidence is required to show the extent of intra-tumor and inter-tumor expression. These studies should focus on the expression patterns of the seven different HLA-G isoforms, as well as the co-localization of HLA-G receptor expression. Finally, which of the HLA-G isoforms play a functional role in cancer immunology must be established.

## Figures and Tables

**Table 1:** Overview of HLA-G-recognizing antibodies and their specificity and applications.

HLA-G mAbs	Specificity	Applications
4H84	An epitope in the HLA-G α1 domain *	IHC(P), IP, WB, ICC, ELISA
MEM-G/1	Denatured HLA-G heavy chain, all isoforms *	IHC(F/P), WB
MEM-G/2	Free heavy chain of all HLA-G isoforms	IHC(F/P), WB
MEM-G/9	Native form of HLA-G1 and HLA-G5 isoform associated with β2M	IHC(F), IP, ELISA, FC
MEM-G/11	HLA-G1	IHC(F), IP, ELISA, FC, ICC
01G	HLA-G1	IHC(F), IP, ICC, FC, ELISA
87G	HLA-G1 and HLA-G5	IHC(F), FC, ELISA
2A12	HLA-G5 and HLA-G6	IHC(F/P), WB, FC, ELISA
5A6G7	HLA-G5 and HLA-G6	IHC(F/P), WB, FC, ELISA, ICC

* Cross-reaction with proteins other than HLA-G has been reported. Abbreviations: Enzyme-Linked Immunosorbent Assay (ELISA), frozen tissue (F), flow cytometry (FC), immunocytochemistry (ICC), immunohistochemistry (IHC), immunoprecipitation (IP), monoclonal antibodies (mAbs), formalin-fixed paraffin-embedded tissue (P), western blot (WB). Adapted from [11].
